# Supplementary material for: MetaRibo-Seq measures translation in microbiomes
Source: Nat Commun. 2020 Jun 29;11:3268. doi: 10.1038/s41467-020-17081-z (PMC7324362; doi:10.1038/s41467-020-17081-z)
Supplement: Supplementary file 10 — Supplementary Data 7 [file 41467_2020_17081_MOESM10_ESM.zip › File2/Confidence_High_Taxonomy/301348_out.krona.html]

Javascript must be enabled to view this page.

members
magnitude
magnitudeUnassigned
count
unassigned
taxon
rank

301348\_out

2

2759
superkingdom
2

2
4751
kingdom

subkingdom
451864
2

phylum
4890
2

2
147537
subphylum

2
class
4891

order
4892
2

2
family
27319

genus
27320
2


SRS1041116\_contig\_number\_1044SRS893366\_contig\_number\_26162
species
73520
2
